# Effects of an Accidental Administration of Ammonia

**Published:** 1853-02

**Authors:** J. H. Beech

**Affiliations:** Coldwater, Mich.


*Effects of an accidental administration of Ammonia.*—The following case, (the report of which is highly creditable to the author's candor and regard for science) will be read with particular interest from its bearing on the question respecting the effect of the ammonia in the cases of supposed death from chloroform reported by Dr. Warren.—Editor Buf. Med. Jour.

*Dr. A. Flint*,

Dear Sir: The circumstances of a double mistake which occurred recently in the surgical clinic of Prof. J. C. Warren, concerning which strictures have appeared from several able sources, have induced me to transmit to you a report of an unfortunate mistake of my own, which may throw, by analogy of symptoms, some light upon the untoward event of Dr. W.'s cases, especially as the first case to which ammonia was not administered, suffered much less than the two with whom it was used.

Mr. C. B., a cooper by trade, aged about twenty-five years, applied to me in April, 1851, having general indisposition with neuralgic symptoms, etc. He said he had felt similarly several times before, and that these symptoms had usually been precursors of severe and protracted illness. After a few prescriptions, the relief from which was only temporary, I suspected verminous irritation, and was strongly supported in the opinion by the patient himself. Accordingly, on the 23d of April, he took at bedtime a full dose of Dolichos Pruriens, and called on me on the morning of the 24th. The bowels had not moved, and I gave him a vial of (as I supposed) Ol. Terebinth, directing him to mix with one tea-spoonful of it, a table-spoonful of castor oil, and take as soon as he reached home. I was summoned hastily in a few minutes and informed that "what I had given him was choking him to death." On my arrival I was shown a half mixed fluid, viz., castor oil and *aqua ammonia*, which I had carelessly given for ol. tere. I might, perhaps, exculpate myself, in part, by farther particulars, but the bare fact is only necessary. The suffocation at first had been extreme. The patient had called for milk as soon as he swallowed the portion, and drank freely. He then irritated the fauces with his finger and emptied the stomach thoroughly. The tongue and tonsils were exceedingly red, the former having been thickly furred before. Respiration was loud and labored. Extreme anxiety of countenance, frequent efforts at deglutition, a short cough evidently restrained as much as possible, and a profuse discharge of ropy mucus from the lungs and throat. The voice was croaking, but soon became more natural. His complaint was of "distress through the chest." The mouth and larynx swelled but slightly. The distress through the lungs was severe, the cough considerable, and the discharge of mucus copious for about thirty-six hours. Both general and local antiphlogistic remedies were promptly used as soon as the system rallied, and on the morning of the 28th, (i. e., the fourth day,) he seemed to have recovered from the effects of the unlucky dose.

About noon of the 23th, the skin was observed to be turning yellow. At 4 o'clock, P. M., the icteric hue was deep, but he was walking about, and said he did not feel very bad. At 10, P. M., I was called, and found him in severe general distress, with high fever, which in about forty-eight hours assumed a low type, during the course of which pneumonia of both sides and, pleuritis in the lower portion of the right thorax were severe complications; and it was not until May 11th, that he was fairly convalescent from a disease, the severity of which I have seldom seen outlived. Pneumonitis was very prevalent in this vicinity at the time.

Private interest, and personal pride, would forbid this exposure; but if in your judgment the facts can be of service, I have no right, or wish, to suppress them. I do not believe the aqua ammonia was the cause of the icterode, or of any part of the disease after the fourth day, (except, perhaps, to increase the pulmonary irritation,) but it was severe enough during the first twenty-four hours to cause fearful forebodings on my part, and would undoubtedly have been much worse had the patient been under the full influence of the safest anæsthetic.

Very respectfully, yours, &c.,

J. H. BEECH, M. D.

COLDWATER, Mich., Jan 18, 1853.

